# THE IMPACT OF PERSISTENT POST-CONCUSSION SYMPTOMS AND EXERCISE INTOLERANCE ON PATIENT-SPECIFIC FUNCTIONING AFTER MILD TRAUMATIC BRAIN INJURY: A BIOPSYCHOSOCIAL PERSPECTIVE

**DOI:** 10.2340/jrm.v58.45811

**Published:** 2026-07-08

**Authors:** Ingerid KLEFFELGÅRD, Lars-Johan Viddal VALAAS, Helene Lundgaard SØBERG, Nada ANDELIC, Cecilie RØE, Sophie STEENSTRUP, Johanna MYHRSTUEN, Tone MARIGÅRD, Lars NYSETHER, Mari Storli RASMUSSEN

**Affiliations:** 1 Department of Physical Medicine and Rehabilitation, Oslo University Hospital HF, Oslo University Hospital, Oslo; 2 University of Oslo, Oslo; 3 Oslo Metropolitan University, Oslo; 4 Sunnaas Rehabilitation Hospital, Bjørnemyr, Norway

**Keywords:** activities of daily living, brain injuries, traumatic, exercise therapy, International Classification of Functioning, disability and health, rehabilitation, signs and symptoms

## Abstract

**Objective:**

To explore activity limitations in a biopsychosocial context in a randomized controlled trial studying sub-symptom threshold aerobic exercise after mild traumatic brain injury.

**Design:**

Qualitative and quantitative approaches.

**Patients:**

Eighty-one participants (58% female; median age 32.3) with persistent post-concussion symptoms and exercise intolerance.

**Methods:**

Activity limitations assessed with the Patient Specific Functional Scale were categorized into main and subcategories using thematic analyses. Subcategories were linked to the International Classification of Functioning, Disability and Health. Group differences and changes over time were examined using linear mixed-effects models.

**Results:**

Of 390 activities, 6 main categories and 24 subcategories were identified: physical activity and exercise (31%), work/education (16%), activities of daily living (15%), social activities (15%), cognition/energy (14%), and sensory-demanding environments (9%). Most subcategories (86%) were linked to the activity and participation domain. There were no group differences in activity limitations at 3 (*p* = 0.60) and 6 (*p* = 0.48) months. However, all patients improved from 3.3 (2.9–3.7) at baseline to 5.4 (5.0–5.9, *p* < 0.001) at 3 months, and 6.2 (5.8–6.7, *p* < 0.001) at 6 months.

**Conclusion:**

Limitations in daily activities were reported across multiple domains. While no differences were found between groups, patient‑reported limitations showed significant improvement over time and may inform individualized rehabilitation strategies.

## LAY ABSTRACT

Many people continue to experience symptoms for months after a mild traumatic brain injury, and these symptoms can make everyday activities difficult. This study explored which activities people found challenging and how these difficulties changed over time. Eighty‑one adults took part about 6 months after their injury. They described activities they struggled with, including exercising, working, studying, socializing, managing daily tasks, thinking clearly, or coping in busy or noisy environments. Most of these challenges were related to everyday activities and participation in daily life. Participants rated how difficult these activities were to perform, and their scores showed meaningful improvement after 3 and 6 months following inclusion in a study evaluating aerobic exercise tailored to avoid worsening symptoms. Overall, the findings highlight the broad impact of mild traumatic brain injury on everyday functioning and emphasize the importance of addressing participation in everyday activities during recovery.

Mild traumatic brain injury (mTBI) is common, with an estimated incidence of 302 per 100,000 person-years in Norway (1). While most patients recover within the first months post-injury, approximately 30–40% experience persisting symptoms beyond 3 months, referred to as persistent post-concussion symptoms (PPCS) (2, 3). PPCS encompass a wide range of difficulties across physical, cognitive, emotional, and social domains, which may substantially affect activities of daily living and quality of life (4, 5).

The presentation and severity of PPCS are heterogeneous, commonly including headache, dizziness, fatigue, and psychological distress (2, 3). There is growing recognition that recovery trajectories following mTBI are complex, highly variable, and influenced by multiple factors like age, sex, prior history of mTBI, and premorbid conditions (6). Exercise intolerance, defined as symptom exacerbation during physical activity and typically confirmed with an incremental exercise test , such as the Buffalo Concussion Treadmill Test (BCTT) (7), has been increasingly recognized in this population, although it has primarily been studied in young athletes (8). Emerging evidence suggests that sub-symptom threshold aerobic exercise may help improve exercise intolerance and support recovery, although its effects on broader aspects of functioning are not yet fully understood (9, 10). Although symptom manifestation varies among patients, exercise intolerance seems to occur across a broad range of post-mTBI symptom profiles (8). Given this complexity, rehabilitation following mTBI requires a multidisciplinary and patient-centred approach that addresses multiple domains of functioning, including physical, psychological, and social health (11, 12).

Standardized patient-reported outcome measures (PROMs) and clinical tests may not fully capture the individual functional limitations most relevant to patients. Patients’ perspectives are essential for understanding post-injury activity limitations and restrictions for developing rehabilitation strategies that are both effective and individually tailored. Incorporating patient-specific measures, such as the Patient-Specific Functional Scale (PSFS) (13), may enhance the evaluation of individual outcomes and provide more meaningful guidance for personalized rehabilitation planning (14, 15). In addition, evaluating patient-identified activity limitations may offer valuable insight into how rehabilitation interventions, such as sub-symptom threshold aerobic exercise, influence everyday functioning over time (14).

The International Classification of Functioning, Disability and Health (ICF) provides a comprehensive framework for assessing functioning across physical, cognitive, emotional, and social domains, while also accounting for environmental and personal factors (16). Greater insight into the health domains reported as sources of functional limitation may yield valuable insights into the types of challenges faced by patients with PPCS and exercise intolerance. This approach supports a holistic perspective in rehabilitation assessment, planning and evaluation. Despite increasing recognition of exercise intolerance as a component of PPCS, limited knowledge exists regarding how these factors influence patients’ everyday functioning and participation within a biopsychosocial context. Hence, the present study had 3 aims:

1. *To explore* perceived activity limitations in patients with PPCS and exercise intolerance after mTBI, using the PSFS.
2. *To map* the activity limitations reported on the PSFS to the ICF and describe the health domains of greatest concern to patients living with PPCS and exercise intolerance.
3. *To assess* group differences and changes in patient-specific activity limitations, measured with the PSFS, at 3 and 6 months following inclusion in a randomized controlled trial (RCT).

## METHODS

This sub-study is part of a single-blind RCT exploring the effects of sub-symptom threshold aerobic exercise in the persistent phase after mTBI. The primary results of the RCT, which showed no between-group differences on the main outcome, the Rivermead Post-Concussion Symptoms Questionnaire, are published elsewhere (9). The RCT is registered in the Clinical Trials Registry (ClinicalTrials.gov: NCT05086419) and approved by the regional ethical committee for Medical and Health Research Ethics (256109) and the Oslo University Hospital (OUH) Data Protection Officer.

### Participants

The study participants were recruited between March 2022 and December 2023 from the outpatient TBI clinic at the Department of Physical Medicine and Rehabilitation at OUH. Inclusion criteria were patients aged 18–60 years with mTBI, PPCS (3 to 24 months post-injury) and self-reported exacerbation of symptoms during physical activity and/or exercise. Patients were excluded if they had severe neurological or psychiatric conditions documented in the medical record, cardiovascular disease or extremity injuries that prevented testing and exercising, or insufficient command of the Norwegian language. They were also excluded if they had a negative test of exercise intolerance (BCTT).

### Procedures and interventions

The recruitment procedures and interventions are described in detail elsewhere (9, 11). Briefly, the treating physician or rehabilitation professionals at the outpatient TBI clinic screened and referred potentially eligible patients to the study physical therapist/researcher (LJVV) who conducted the baseline assessments. The baseline assessment included patients completing patient-reported outcome measures (PROMs), participating in an interview regarding problems in functioning on the PSFS, and performing the exercise intolerance test (BCTT). All patients were assessed prior to group allocation in the RCT, and eligible patients were subsequently included in the study and randomized to the intervention or control group. Both groups received the usual multidisciplinary outpatient rehabilitation provided at OUH. Patients allocated to the intervention group completed a 12-week sub-symptom threshold aerobic exercise programme that was individually adapted with supervision and follow-ups every third week. Patients allocated to the control group received general advice regarding physical activity based on recommendations from the Norwegian Directorate of Health (9, 17). In the current study, patients in both the intervention and control group were included. Follow-up assessments were performed at 3 and 6 months.

### Data and outcome measures

Demographic data and injury-related factors were collected from the patient’s medical records and during the baseline assessments.

The PSFS was used to assess the patient’s self-perceived activity limitations. The PSFS (13) is a patient-specific outcome measure in which patients identify 3–5 activities they are unable to perform or have difficulty performing due to their PPCS after mTBI. Patients rate the current level of difficulty associated with each activity using a Numeric Rating Scale (NRS) ranging from 0 (unable to perform the activity) to 10 (able to perform the activity with no difficulty or as before the injury). The PSFS therefore provides text-based descriptions of functional limitations for analysis in addition to numerical data. At the follow-up assessments, the patients were asked if they still had difficulties with the activities listed at the baseline assessment and were asked to re-rate the level of difficulty on the NRS scale. The mean score of the reported PSFS items scores is calculated, and a change of ≥ 2 is considered a clinically and statistically relevant change (14, 15).

### Data analysis

The PSFS rendered data analysed by qualitative and quantitative approaches. The text data were analysed in 2 steps similar to methods proved to be feasible in an earlier study by Valovich McLeod et al. (18); first by applying elements from thematic analysis to identify patterns within the reported activities (19, 20), and second by linking these thematic categories to the ICF, applying the ICF linking rules (21). In this process, patient-specific activities were organized into main categories and subcategories by a 4-person research team consisting of 2 senior researchers and physiotherapists (MR and IK), and 2 junior researchers (physiotherapist JM and occupational therapist TM). Thematic analysis is a method used to analyse qualitative data and involves several steps including reading, coding, and interpretation of the data (19, 20). In the current study the text data were brief sentences of functional limitations; however, we used the following steps as suggested by Braun and Clarke: (*i*) Familiarization with the data: The responses from the PSFS were read through systematically to get an overall sense of the content. (*ii*) Initial coding: Initial impressions and recurrent activities were noted. (*iii*) Collate codes with supporting data: The activities were assigned codes capturing the essence of the content. Similar codes were grouped to form subcategories representing different aspects of the data. (*iv*) Generate themes: The subcategories were synthesized into broader main categories that reflect the overarching patterns in the data. (*v*) Review and refine main categories: The main categories were evaluated to ensure that they accurately reflected the data and were distinct from each other. (*vi*) Define and name main categories: The main categories were given a descriptive and informative name (19, 20).

To preserve the specificity of participants’ perceived activity limitations and problems in functioning, the linking to the ICF was conducted at the subcategory level of the qualitative analysis, in accordance with established ICF linking rules (21). The linking was performed by the same 4-person research team in 2 steps. First the linking was done separately by (*i*) a physiotherapist/researcher (IK) and (*ii*) a physiotherapist (JM) and an occupational therapist (TM). Subsequently, disagreements in linking were discussed, and consensus was reached in cooperation with a third physiotherapist/researcher (MSR).

### Statistical analyses

Descriptive statistics were used to summarize baseline demographics, injury-related factors, and PSFS scores for all main and subcategories. Normality was assessed using visual inspection of histograms and Q–Q plots. Normally distributed data are presented as mean and standard deviation (SD) whereas non-normally distributed data with low n are presented as median and interquartile range (p25–p75).

The between-group differences for the PSFS scores were estimated from baseline to 3 and 6 months by a repeated measures linear mixed-effect model (LMM) with maximum likelihood (ML) estimation, accounting for intra-individual correlations over time.

Changes in patient‑specific activity limitations for all patients were analysed for both the overall PSFS score and for each main category using LMMs with a random intercept for each participant. Assumptions were verified by visual inspection of scatterplots and examination of residual plots. In the overall model, assessing the total PSFS score, time, group, age, sex, and time since injury were included as fixed effects. In the model examining the PSFS scores for the main categories, the main category was added as an additional fixed effect. Group, age, sex, and months since injury were treated as covariates. Age and sex were included as standard confounders. Group allocation accounted for differences between intervention arms, and time since injury was included to adjust for variability in recovery trajectories, as participants were enrolled between 3 months and 2 years post-injury. All models were estimated using maximum likelihood. Adjusted marginal means with 95% confidence intervals were calculated, and pairwise comparisons across time points were corrected using Holm’s method. The Statistical Package for the Social Sciences (SPSS) version 30.0 was used for descriptive statistics (IBM Corp, Armonk NY, USA). Repeated measure LMMs were conducted in STATA v. 17 (StataCorp LLC, College Station, TX, USA) using “meglm” and “metobit” commands, respectively, with constraints applied to adjust for baseline values. Statistical analyses of the PSFS scores were conducted using RStudio (v.2025.02.2; R Foundation for Statistical Computing, Vienna, Austria).

## RESULTS

A total of 81 patients diagnosed with mTBI, 47 (58%) females, median age 32.3 years (P25–P75 29.3–43.5) with PPCS and exercise intolerance were included in this study at approximately 6 months post-injury. Demographic and injury related data are presented in Table I.

**Table I:** Demographic and injury-related variables (*n* = 81)

Demographic or injury-related variable	Value
Age (years), median (p25–p75)	32.3 (29.3–43.5)
Female, *n* (%)	47 (58)
Marital status, *n* (%)	
Married/cohabitating	41 (51)
Single/divorced	37 (45)
Other	3 (4)
Education in years, mean (SD)	17.7 (3.5)
Employment status pre-injury, *n* (%)	
Employed/studying	77 (95.1)
Unemployed/social support	4 (4.9)
Post-injury sick leave status, *n* (%)	
Non/partial	43 (52.5)
Full time	38 (47.5)
Days since injury, median (p25–p75)	182 (137–248.5)
GCS, median (p25–p75), *n* = 66	15 (15–15)
Complicated, *n* (%)	
Positive CT/MRI scan	2 (2.5)
Negative CT/MRI scan/unknown	79 (97.5)
Cause of injury, *n* (%)	
Fall	27 (33.3)
Bike and traffic	17 (21.0)
Sport	14 (17.3)
Violence	4 (4.9)
Other	19 (23.5)

*n*: sample size; p: percentile; SD: standard deviation; CT: computed tomography; MRI: magnetic resonance imaging.

The adjusted mean difference on the PSFS score between intervention and control groups at 3 and 6 months were 0.21, *p* = 0.60 and 0.30, *p* = 0.48 respectively (Table II). As no between‑group differences were found between the intervention and control groups, the groups were merged for the analyses of the PSFS outcomes.

**Table II:** PSFS scores and between-group differences from baseline to 3 and 6 months

Time	Intervention group Adjusted mean (CI)	Control group Adjusted mean (CI)	Adjusted mean difference between groups	*p*-value
Baseline	3.27 (2.87–3.67)*	3.27 (2.87–3.67)*		
3 months	5.52 (4.93–6.11)	5.31 (4.73–5.89)	0.21 (–0.56 to 0.98)	0.60
6 months	6.38 (5.76–6.99)	6.08 (5.47–6.69)	0.30 –0.52 to 1.11)	0.48

* Adjustment for baseline values eliminated baseline differences between groups.

**Fig. 1:**
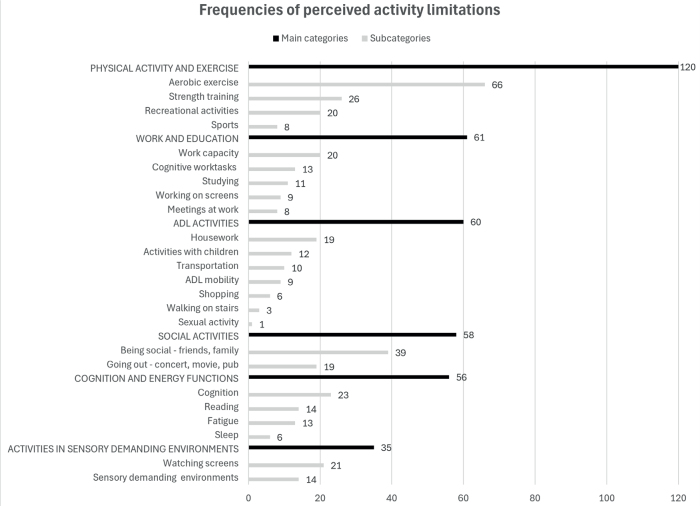
Main categories (in black) with corresponding subcategories (in grey) from the PSFS. Numbers represent the actual count of reported activities within each category. ADL: activities of daily living.

Regarding the PSFS the 81 patients nominated 390 activities at baseline, which were grouped into 6 main categories comprising 24 subcategories. The most frequently nominated main category was physical activity and exercise (31%) followed by work and education (16%), activities of daily living (15%), social activities (15%), cognition and energy functions (14%), and activities in sensory demanding environments (9%) (Fig. 1 and Table III). The main category rated as most difficult was activities in sensory demanding environments followed by work and education and physical activity and exercise. Activities of daily living were reported as the least difficult main category (Table III).

**Table III:** Mean and median PSFS scores (0–10, unable–able to perform the activity) for the main and subcategories at baseline

Main category	^†^ Mean PSFS score per main category (SD)	Subcategory	Median PSFS score per subcategory (p25–p75)
Physical activity and exercise	2.88 (2.16)	Aerobic exercise (running, swimming, jogging, walking, biking/spinning, group training, cross-country skiing)	3 (1–5)
		Strength training	3 (2–4)
		Recreational activities (dancing, mountain skiing, hiking hills, hiking mountains, rowing a boat, kayaking, playing piano, gardening, activities with increased gravity like waterjets)	3.5 (0.5–6.5)
		Sports (tennis, football, handball, ice hockey, sand volleyball, paddle tennis)	1 (0–5.5)
Work and education	2.83 (2.03)	Work capacity	2.5 (0.5–4.5)
		Cognitive task at work	3 (2–4)
		Working on screens	2 (0.5–3.5)
		Meetings at work	2 (2–3)
		Studying	4 (2.5–5.5)
ADL activities	4.36 (1.83)	Housework (cleaning, cooking)	5 (4–6)
		Activities with children (playing, carrying, baking, taking to activities, reading)	5 (3–5)
		Transportation (public, biking in city, driving)	3 (2–6)
		Mobility (lifting, carrying, bending)	3 (3–5)
		Shopping	4 (3–5)
		Walking down steep stairs/walking downstairs	4 (3.5–4)
		Sexual activity	4 (4–4)
Social activities	3.52 (1.85)	Being social for more than 1 hour (with several people, dinner with friends, dinner party, eating with colleagues, party with music, meeting friends)	4 (2–4.5)
		Going out (to a café, party, pub, to the city, concerts, cultural events, shows, shopping malls, galleries, movies)	2 (1–4.5)
Cognition and energy functions	3.56 (1.69)	Cognition (remembering, concentration, receiving information, analytic thinking, keeping appointments, conversations/communication, getting things done, organizing and planning activities, thinking slower, problem solving, looking/searching for things)	3.5 (2.5–5)
		Reading	3 (2–4)
		Fatigue (capacity dual/multi-tasking)	3 (2–3)
		Sleep	4 (4–6)
Activities in sensory demanding environments	2.76 (1.57)	Watching screens	2 (1–4)
		Sensory demanding environments (noise/light sensitivity)	2 (2–3)

† If 1 patient scored same categories multiple times, average score per patient.

PSFS: Patient Specific Functional Scale; SD: standard deviation.

The subcategories most frequently reported by ≥ 20 patients were: aerobic exercise (66), being social (39), strength training (26), cognition (23), watching screens (21), work capacity (20), recreational activities (20) (see Fig. 1 and Table III).

The difficulty in performing the activities in each subcategory at baseline varied from 0 to 9 with median PSFS scores ranging from 1 to 5. The subcategory rated as most difficult to perform at baseline was different kinds of sport activities (median 1, p25–p75: 0–5.5). The subcategories reported least difficult were housework (median 5, p25–p75: 4–6) and activities with children (median 5, p25–p75: 3–5) (see Table III).

The subcategories were linked to the ICF, with most (86%) linked to the domains of “Activities and participation”; 11% were linked to the “Body function” domain, and 3% were linked to “Environmental factors”.

The subcategories represented 10 different ICF chapters, including “Mental functions” in the body structure and function domain of the ICF to “Community, social and civic life”, “Major life areas”, and “Domestic life and mobility” in the activities and participation domain of ICF. In total, 32 ICF categories were represented, with “Recreation and leisure” being the most frequent category (Table SI).

The adjusted mean total PSFS scores increased from 3.26 (95% CI 2.85–3.67) at baseline to 5.41 (95% CI 4.96–5.86, *p* < 0.001) at 3 months and 6.22 (95% CI 5.75–6.69, *p* < 0.001) at 6 months (Table SII). Linear mixed effects models indicated that the adjusted mean change scores for both time intervals were statistically significant (*p* < 0.001). Pairwise improvements from baseline to 3 months (β = 2.15, SE = 0.22) and 6 months (β = 2.96, SE = 0.23) were statistically significant (*p* < 0.001), with no significant covariates (group [*p* = 0.727]; age [*p* = 0.320], sex [*p* = 0.976], time since injury [*p* = 0.442]) (Table SII).

**Fig. 2:**
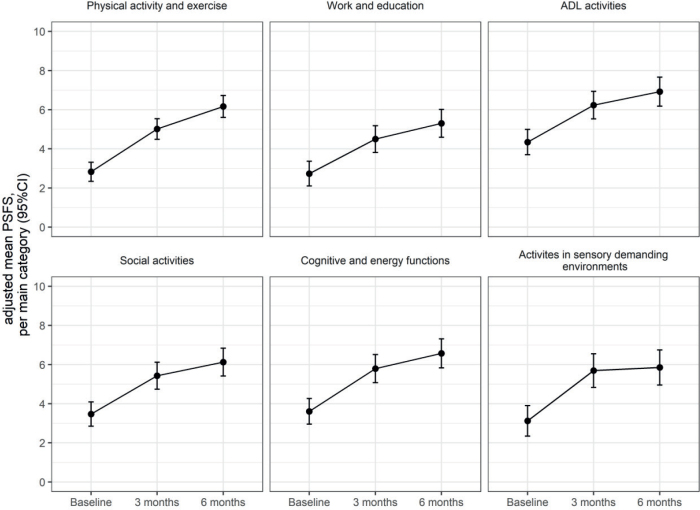
Change in Patient Specific Functional Scale (PSFS) scores for each main category from baseline to 3 and 6 months. CI: confidence interval.

From baseline to 3 months and from baseline to 6 months, all main categories showed significant improvement. Each category also improved significantly between 3 and 6 months, although the changes were smaller during this later period. The largest improvement was seen in physical activity and exercise (β = 3.3, SE = 0.30, *p* < 0.001), while the smallest improvement occurred in work and education (β = 2.59, SE = 0.41, *p* < 0.001) (Fig. 2).

## DISCUSSION

This study demonstrates that individuals with PPCS accompanied by exercise intolerance experience a broad spectrum of activity limitations that affect multiple aspects of daily life following mTBI. The patient-nominated activities predominantly reflected restrictions within the “Activities and participation” domain of the ICF, with limitations related to physical activity and exercise reported most frequently. All participants demonstrated statistically and clinically meaningful improvements over the course of this study, which aimed to promote physical activity and exercise after mTBI. However, no added effects of the RCT intervention were observed. This pattern is consistent with the findings from the other patient-reported outcome measures in the RCT study (9).

To our knowledge, this is the first study to report on activity limitations in adult patients living with PPCS and exercise intolerance after mTBI. The high frequency of activity limitations related to physical activity and exercise should be interpreted in light of the study context. Because data were collected within a clinical trial focusing on aerobic exercise, where participants self-reported exacerbation of symptoms during physical activity and/or exercise, they may have been more attuned to limitations related to these activities. Furthermore, this is particularly relevant for individuals with persistent symptoms after mTBI, as difficulties with physical activity and exercise are common in this population (8, 22). As a result, activities directly related to physical activity and exercise may have been prioritized over other challenges that are nonetheless clinically important but less likely to be spontaneously reported. Future studies would also benefit from comparing patients with PPCS who exhibit exercise intolerance with those who do not, as this may help clarify whether activity-related limitations differ between these subgroups.

Our study also identified limitations across several other aspects of life, including work and education, activities of daily living (ADL), social participation, cognitive functioning, and activities in sensory-demanding environments. These results are consistent with previous studies demonstrating that patients with PPCS often experience restrictions across multiple aspects of functioning, including social roles, occupational and educational participation, ADL functioning, and cognitive activities (6, 23–25). Overall, our findings point to the need for rehabilitation strategies that extend beyond a narrow focus on physical activity and exercise. Instead, a more comprehensive, multidimensional approach is warranted that addresses the diverse activity limitations reported by patients across physical, cognitive, social, and environmental domains (6). However, recent shifts in clinical recommendations – from prolonged rest until full symptom resolution to earlier resumption of activity – suggest that incorporating aerobic exercise may be beneficial as part of rehabilitation following mTBI (26–30). Such an approach not only facilitates gradual physical re-engagement but may also facilitate patients’ return to meaningful activities, potentially preventing the vicious circle of inactivity and deconditioning often seen in this population (31, 32).This is particularly relevant given that many participants in our study identified physical activity and exercise themselves as key activity limitations. Although emerging evidence highlights the potential role of aerobic activity in mTBI rehabilitation, our findings did not show differential benefits of the programme on patient-specific functioning or activity limitations. This may be due to the similar levels of physical activity observed in both groups in our RCT. Furthermore, all participants were tested for exercise intolerance and received general advice concerning physical activity (9).

With regard to the PSFS scores, the overall mean baseline rating was 3.3 points, positioned below the midpoint of the scale between “unable to perform the activity” and “able to perform the activity without difficulty or as before the injury”. This indicates that participants reported moderate to severe limitations in their identified activities at baseline. Most participants did not report on activities they were entirely unable to do. Instead, in accordance with previous studies (33, 34), they primarily identified problems with activities that were difficult to perform or that required modification.

Activities in sensory‑demanding environments were rated as the most difficult, particularly those involving screens, noise, and bright light. These symptoms are well recognized after brain injuries (35, 36) and reflect disruptions in sensory‑gating mechanisms that reduce the brain’s ability to filter visual and auditory input (37). As a result, everyday stimuli such as digital screens, fluorescent lighting, and background noise can trigger headaches, dizziness, and cognitive fatigue (37). Such sensitivities can substantially limit participation in work, social, and daily activities (35). The improvements observed in our cohort suggest that sensory intolerance may be responsive to rehabilitation strategies that gradually increase sensory tolerance and support autonomic regulation.

Work and education were rated as the second most difficult activities, which is consistent with evidence showing that returning to cognitively demanding, time pressured, and socially complex environments is one of the greatest challenges after mTBI (12, 38–40). These tasks typically require sustained attention, rapid information processing, multitasking, and tolerance of sensory stimulation, domains commonly affected in individuals with PPCS, which was also confirmed in this study. Activities of daily living were reported as the least difficult, suggesting that basic functional abilities are less affected at this stage of recovery, whereas higher level activities place greater demands on cognitive and physical endurance.

From baseline to 3 months, the mean overall PSFS change surpassed the minimal detectable change of 2 points and equalled the minimal clinically important difference of 2.2 points reported in previous studies (13, 15, 41). This suggests that the observed improvements not only represented a relevant statistical change but also a clinically relevant change. By 6 months, these improvements had progressed further, although the magnitude of change from 3 to 6 months was less pronounced. Furthermore, all main categories showed significant improvement from baseline to 3 months and again to 6 months, indicating broad functional gains. The largest improvement was seen in the physical activity and exercise category, which may reflect both the focus of the study and the guidance provided to all participants to gradually resume physical activity. In contrast, the work and education category showed the smallest improvement across timepoints, which reflects the need for environmental accommodations, and the cumulative cognitive load associated with returning to work or study (12, 38–40). The clinically meaningful change during this phase underscores the potential for continued functional recovery and adaptation-provided treatment addressing individual rehabilitation needs and targeted support for work and education. However, the results also highlight that improvements in physical activity may not necessarily translate into equivalent gains in more complex participation domains.

All identified subcategories could be linked to the ICF framework, with the majority mapping to the domains of activities and participation. This pattern aligns with previous studies (18, 34), and reflects the structure of the PSFS, which explicitly asks patients to identify activities they find difficult to perform. While many standardized PROMs primarily assess symptoms and body functions, the PSFS serves as a valuable complement by capturing patient-identified limitations in real-world activities. In this way, the PSFS highlights specific and individualized challenges faced by patients with PPCS and exercise intolerance after mTBI, providing clinically relevant information that might otherwise be overlooked. By integrating patient-identified activity limitations from the PSFS within the ICF framework, rehabilitation efforts can more effectively target the domains of functioning that are most meaningful to patients. This alignment not only strengthens the patient-centred nature of assessment but also provides a structured foundation for tailoring individualized rehabilitation strategies.

### Limitations

This study has some limitations that should be acknowledged. The patients were recruited from a university hospital and represent a sample of individuals with long-lasting PPCS and exercise intolerance referred to an outpatient TBI clinic. Additionally, approximately 20% of the invited patients were excluded due to a negative BCTT, indicating no exercise intolerance. However, the sex distribution and median age of the included participants were comparable to those of other patients with PPCS referred to the clinic (42), supporting the overall generalizability of the findings to mTBI populations typically seen in university hospital settings. Although participants had a higher mean educational level than the general population in Norway, this likely reflects the higher educational attainment within the OUH catchment area and the typical demographic profile of patients referred to our clinic (42). Nevertheless, this characteristic may limit the generalizability of the findings to populations and clinical settings with different sociodemographic profiles.

### Conclusion

This study demonstrates that patients with PPCS and exercise intolerance after mTBI experience activity limitations across multiple domains of daily life. Most reported restrictions were linked to the ICF domains of activities and participation, with physical activity and exercise emerging as the most frequently affected categories. Changes in perceived activity limitations from inclusion in this study to 3 and 6 months were clinically meaningful and statistically significant. These findings underscore the importance of assessing patient-identified activity limitations to better capture the breadth and complexity of challenges experienced by individuals with PPCS. Incorporating such patient-derived insights into rehabilitation planning may facilitate the development of more targeted, individualized, and patient-centred interventions.

## Supplementary Material



## References

[1] Skandsen T, Nilsen TL, Einarsen C, Normann I, McDonagh D, Haberg AK, et al. Incidence of mild traumatic brain injury: a prospective hospital, emergency room and general practitioner-based study. Front Neurol 2019; 10: 638. 10.3389/fneur.2019.00638 31275229 PMC6591366

[2] Voormolen DC, Haagsma JA, Polinder S, Maas AIR, Steyerberg EW, Vuleković P, et al. Post-concussion symptoms in complicated vs. uncomplicated mild traumatic brain injury patients at three and six months post-injury: results from the CENTER-TBI Study. J Clin Med 2019; 8. 10.3390/jcm8111921 31717436 PMC6912209

[3] Machamer J, Temkin N, Dikmen S, Nelson LD, Barber J, Hwang P, et al. Symptom frequency and persistence in the first year after traumatic brain injury: a TRACK-TBI study. J Neurotrauma 2022; 39: 358–370. 10.1089/neu.2021.0348 35078327 PMC8892966

[4] Voormolen DC, Polinder S, von Steinbuechel N, Vos PE, Cnossen MC, Haagsma JA. The association between post-concussion symptoms and health-related quality of life in patients with mild traumatic brain injury. Injury 2019; 50: 1068–1074. 10.1016/j.injury.2018.12.002 30554897

[5] Sveen U, Ostensjo S, Laxe S, Soberg HL. Problems in functioning after a mild traumatic brain injury within the ICF framework: the patient perspective using focus groups. Disabil Rehabil 2013; 35: 749–757. 10.3109/09638288.2012.707741 22897238

[6] King S, Winkens I, Wijenberg M, Schepers J, Stapert S, Verbunt J, et al. Recovery trajectories of patients with mild traumatic brain injury. J Neurotrauma 2025; 42: 1345–1358. 10.1089/neu.2024.0610 40432603

[7] Kozlowski KF, Graham J, Leddy JJ, Devinney-Boymel L, Willer BS. Exercise intolerance in individuals with postconcussion syndrome. J Athl Train 2013; 48: 627–635. 10.4085/1062-6050-48.5.02 23952041 PMC3784364

[8] Antonellis P, Campbell KR, Wilhelm JL, Shaw JD, Chesnutt JC, King LA. Exercise intolerance after mild traumatic brain injury occurs in all subtypes in the adult population. J Neurotrauma 2024; 41: 635–645. 10.1089/neu.2023.0168 37534853 PMC11071083

[9] Valaas LV, Soberg HL, Rasmussen MS, Steenstrup SE, Brunborg C, Røe C, et al. Effects of sub-symptom threshold aerobic exercise on persistent postconcussion symptom burden and exercise intolerance: a randomized controlled trial. Phys Ther 2026; 10.1093/ptj/pzag049. 10.1093/ptj/pzag049 PMC13252595 42113627

[10] Mercier LJ, McIntosh SJ, Boucher C, Joyce JM, Batycky J, Galarneau JM, et al. Effect of aerobic exercise on symptom burden and quality of life in adults with persisting post-concussive symptoms: the ACTBI randomized controlled trial. Arch Phys Med Rehabil 2025; 106: 195–205. 10.1016/j.apmr.2024.10.002 39427780

[11] Valaas LV, Soberg HL, Rasmussen MS, Steenstrup SE, Andelic N, Kleffelgård I. Sub-symptom threshold aerobic exercise for patients with persisting post-concussion symptoms and exercise intolerance after mild traumatic brain injury: a study protocol with a nested feasibility study for a randomized controlled trial. BMC Neurol 2023; 23: 179. 10.1186/s12883-023-03221-7 37138202 PMC10155435

[12] Fure SCR, Howe EI, Andelic N, Brunborg C, Sveen U, Røe C, et al. Cognitive and vocational rehabilitation after mild-to-moderate traumatic brain injury: a randomised controlled trial. Ann Phys Rehabil Med 2021; 64: 101538. 10.1016/j.rehab.2021.101538 33957293

[13] Stratford P, Gill C, Westaway M, Binkley J. Assessing disability and change on individual patients: a report of a patient specific measure. Physiotherapy Canada 1995; 47: 258–263. 10.3138/ptc.47.4.258

[14] Evensen J, Soberg HL, Sveen U, Hestad KA, Bronken BA. The applicability of the patient-specific functional scale (PSFS) in rehabilitation for patients with acquired brain injury (ABI): a cohort study. J Multidiscip Healthc 2020; 13: 1121–1132. 10.2147/jmdh.S259151 33116558 PMC7553661

[15] Evensen J, Soberg HL, Sveen U, Hestad KA, Moore JL, Bronken BA. Measurement properties of the patient-specific functional scale in rehabilitation for patients with stroke: a prospective observational study. Phys Ther 2023; 103. 10.1093/ptj/pzad014 PMC10158643 37140476

[16] International Classification of Functioning DaHI. 2018 [cited 2026 May 22]. Available from: https://www.who.int/standards/classifications/international-classification-of-functioning-disability-and-health

[17] Norwegian Directorate of Health. National Professional Guidelines for Physical Activity in Prevention and Treatment. 2019 [cited 2026 May 22]. Available from: https://www.helsedirektoratet.no/faglige-rad/fysisk-aktivitet-i-forebygging-og-behandling

[18] Valovich McLeod TC, Williams RM, Snyder Valier AR. The adolescent patient perspective on activity limitations after sport-related concussion. J Athl Train 2024; 59: 984–990. 10.4085/1062-6050-0587.23 38477112 PMC11537216

[19] Terry G, Hayfield N, Clarke V, Braun V. Thematic analysis. In: Willig C, Rogers WS. The SAGE handbook of qualitative research in psychology,. Thousand Oaks, CA/London: Sage Publications; 2017. Ch 2, p. 25.

[20] Braun V, Clarke V. Reflecting on reflexive thematic analysis. Qual Res Sport Exerc Health 2019; 11: 589–597.

[21] Cieza A, Fayed N, Bickenbach J, Prodinger B. Refinements of the ICF Linking Rules to strengthen their potential for establishing comparability of health information. Disabil Rehabil 2019; 41: 574–583. 10.3109/09638288.2016.1145258 26984720

[22] Lee Y, Choi Y, Jeon J, Leigh JH, Kim DK, Oh BM. Impact of mild traumatic brain injury on health behaviors. Sci Rep 2025; 15: 1585. 10.1038/s41598-024-83920-4 39794413 PMC11723977

[23] Andelic N, Røe C, Tenovuo O, Azouvi P, Dawes H, Majdan M, et al. Unmet rehabilitation needs after traumatic brain injury across Europe: results from the CENTER-TBI study. J Clin Med 2021; 10. 10.3390/jcm10051035 33802336 PMC7959119

[24] Howe EI, Zeldovich M, Andelic N, von Steinbuechel N, Fure SCR, Borgen IMH, et al. Rehabilitation and outcomes after complicated vs uncomplicated mild TBI: results from the CENTER-TBI study. BMC Health Serv Res 2022; 22: 1536. 10.1186/s12913-022-08908-0 36527074 PMC9758851

[25] Carroll LJ, Cassidy JD, Cancelliere C, Cote P, Hincapie CA, Kristman VL, et al. Systematic review of the prognosis after mild traumatic brain injury in adults: cognitive, psychiatric, and mortality outcomes: results of the International Collaboration on Mild Traumatic Brain Injury Prognosis. Arch Phys Med Rehabil 2014; 95: S152–173. 10.1016/j.apmr.2013.08.300 24581903

[26] Leddy JJ, Haider MN, Ellis M, Willer BS. Exercise is medicine for concussion. Curr Sports Med Rep 2018; 17: 262–270. 10.1249/jsr.0000000000000505 30095546 PMC6089233

[27] Silverberg ND, Otamendi T. Advice to rest for more than 2 days after mild traumatic brain injury is associated with delayed return to productivity: a case-control study. Front Neurol 2019; 10: 362. 10.3389/fneur.2019.00362 31037065 PMC6476280

[28] Silverberg ND, Iaccarino MA, Panenka WJ, Iverson GL, McCulloch KL, Dams-O’Connor K, et al. Management of concussion and mild traumatic brain injury: a synthesis of practice guidelines. Arch Phys Med Rehabil 2020; 101: 382–393. 10.1016/j.apmr.2019.10.179 31654620

[29] Silverberg ND, Leddy JJ. Progress in concussion/traumatic brain injury science and clinical care over the last 40 years. J Head Trauma Rehabil 2026; 41: 2–15. 10.1097/htr.0000000000001102 40853263

[30] Rytter HM, Graff HJ, Henriksen HK, Aaen N, Hartvigsen J, Hoegh M, et al. Nonpharmacological treatment of persistent postconcussion symptoms in adults: a systematic review and meta-analysis and guideline recommendation. JAMA Netw Open 2021; 4: e2132221. 10.1001/jamanetworkopen.2021.32221 34751759 PMC8579233

[31] Silverberg ND, Iverson GL. Is rest after concussion “the best medicine?”: recommendations for activity resumption following concussion in athletes, civilians, and military service members. J Head Trauma Rehabil 2013; 28: 250–259. 10.1097/HTR.0b013e31825ad658 22688215

[32] Quatman-Yates CC, Hunter-Giordano A, Shimamura KK, Landel R, Alsalaheen BA, Hanke TA, et al. Physical therapy evaluation and treatment after concussion/mild traumatic brain injury. J Orthop Sports Phys Ther 2020; 50: Cpg1–cpg73. 10.2519/jospt.2020.0301 32241234

[33] Maskell F, Chiarelli P, Isles R. Dizziness after traumatic brain injury: results from an interview study. Brain Inj 2007; 21: 741–752. 10.1080/02699050701472109 17653948

[34] Storløs B, Roaldsen KS, Soberg HL, Kleffelgaard I. Patient-specific functioning related to dizziness and balance problems after traumatic brain injury: a cross sectional study using an ICF perspective. Cogent Med 2021; 8: 1932247. 10.1080/2331205X.2021.1932247

[35] de Sain AM, Pellikaan LWM, van Voskuilen J, Migdis M, Sommers-Spijkerman MPJ, Visser-Meily JMA, et al. Sensory hypersensitivity after acquired brain injury: the patient perspective. Disabil Rehabil 2024; 46: 3586–3593. 10.1080/09638288.2023.2251401 37649314

[36] Shepherd D, Landon J, Kalloor M, Barker-Collo S, Starkey N, Jones K, et al. The association between health-related quality of life and noise or light sensitivity in survivors of a mild traumatic brain injury. Qual Life Res 2020; 29: 665–672. 10.1007/s11136-019-02346-y 31667708

[37] Kumar S, Rao SL, Nair RG, Pillai S, Chandramouli BA, Subbakrishna DK. Sensory gating impairment in development of post-concussive symptoms in mild head injury. Psychiatry Clin Neurosci 2005; 59: 466–472. 10.1111/j.1440-1819.2005.01400.x 16048453

[38] Howe EI, Fure SCR, Løvstad M, Enehaug H, Sagstad K, Hellstrøm T, et al. Effectiveness of combining compensatory cognitive training and vocational intervention vs. treatment as usual on return to work following mild-to-moderate traumatic brain injury: interim analysis at 3 and 6 month follow-up. Front Neurol 2020; 11: 561400. 10.3389/fneur.2020.561400 33240196 PMC7683428

[39] Petty J, McLennan V, Kendall E, Degeneffe CE. Scoping review of return-to-work interventions for persons with traumatic brain injury. Disabil Rehabil 2024; 46: 3243–3255. 10.1080/09638288.2023.2243583 37551864

[40] Spjelkavik Ø, Enehaug H, Klethagen P, Howe EI, Fure SCR, Terjesen HCA, et al. Workplace accommodation in return to work after mild traumatic brain injury. Work 2023; 74: 1149–1163. 10.3233/wor-211440 36442182

[41] Young IA, Cleland JA, Michener LA, Brown C. Reliability, construct validity, and responsiveness of the neck disability index, patient-specific functional scale, and numeric pain rating scale in patients with cervical radiculopathy. Am J Phys Med Rehabil 2010; 89: 831–839. 10.1097/PHM.0b013e3181ec98e6 20657263

[42] Hovset CG, Røe C, Søberg HL, Brunborg C, Helseth E, Andelic N, et al. Patient satisfac-tion with rehabilitation services following traumatic brain injury: a quality registry study. J Rehabil Med 2024; 56: jrm35115. 10.2340/jrm.v56.35115 39539069 PMC11577624

