# Supplementary material for: THE IMPACT OF PERSISTENT POST-CONCUSSION SYMPTOMS AND EXERCISE INTOLERANCE ON PATIENT-SPECIFIC FUNCTIONING AFTER MILD TRAUMATIC BRAIN INJURY: A BIOPSYCHOSOCIAL PERSPECTIVE
Source: J Rehabil Med. 2026 Jul 8;58:45811. doi: 10.2340/jrm.v58.45811 (PMC13355155; doi:10.2340/jrm.v58.45811)
Supplement: Supplementary file 1 [file JRM-58-45811-s1.pdf]

Table SI: The ICF linking of the PSFS subcategories to ICF chapters and categories.

ICF domain, n (%)	PSFS main category	PSFS subcategory (frequency)	ICF chapter	ICF category
Body structure and function<br><br>42 (11%)	Cognition and energy functions	Cognition (23)	Mental functions	Attention functions (b140), memory functions (b144), thought functions (b160), higher-level cognitive functions (b164), mental functions of language (b167), mental functions, other specified (b198)
		Sleep (6)	Mental functions	Sleep (b134)
		Fatigue/ capacity dual/multi-tasking (13)	Mental functions	Energy and drive functions (b130)
Activities and participation<br><br>334 (86%)	Physical activity and exercise	Aerobic exercise (66)	Community, social and civic life / walking and moving	Recreation and leisure (d920), moving around (d455)
		Strength training (26)	Community, social and civic life	Recreation and leisure (d920)
		Sports (8)	Community, social and civic life	Recreation and leisure (d920)
		Recreational activities (20)	Community, social and civic life/walking and moving	Recreation and leisure (d920) walking (d450)
	Work and education	Working including Work capacity, meetings at work, cognitive task at work, working on screens (50)	Major life areas	Remunerative employment (d850)
		Studying (11)	Major life areas	Higher education (d830)
	Domestic and ADL-activities	Housework, including cleaning, cooking (19)	Domestic life	Doing housework (d640) preparing meals (d630)
		Activities with children – (12) playing, carrying, baking, taking to activities, reading	Domestic life	Assisting others (d660)
		Transportation – public, biking in city, driving (10)	Mobility	Moving around using transportation (d470), driving (d475)
		Lifting, carrying, bending – mobility (9)	Mobility	Lifting and carrying objects (d430)<br>Changing basic body position (d410)
		Shopping (6)	Domestic life	Acquisition of goods and services (d620)
		Walking up/downstairs (3)	Mobility	Going up and down stairs (d451)
		Sexual activity (1)	Interpersonal interactions and relationships	Intimate relationships (d770)
	Social activities	Being social (39)	Interpersonal interactions and relationships	Particular interpersonal relationships, other specified and unspecified (d779), relating with strangers (d730), formal relationships (d740), informal social relationships (d750), family relationships (d760)
		Going out (19)	Community, social and civic life	Recreational and leisure (d920)

	Cognition and energy functions	Reading (14)	Learning and applying knowledge	Applying knowledge (d166)
	Activities in sensory demanding environments	Watching screens (21)	Learning and applying knowledge	Watching (d110)
Environmental factors 14 (3%)	Activities in sensory demanding environments	Noisy environment and Environments with bright lights/light sensory demanding environments (14)	Natural environment and human-made changes to environment	Sound (e250)<br>Light (e240)

Abbreviations: ICF: International Classification of Functioning, Disability and Health, PSFS: Patient Specific Functional Scale, ADL: Activities of Daily Living.

Table SII: The PSFS scores (0-10, unable - able to perform the activity) for the PSFS total score and for the main categories at baseline, 3 and 6 months.

Activity	Baseline (T0)	3 months (T1)	6 months (T2)	T0-T1	T0-T2	T1-T2
	Adjusted mean (β) [95% CI]	Adjusted mean (β) [95% CI]	Adjusted mean (β) [95% CI]	p	p	p
PSFS total score	3.26 [2.85 to 3.67]	5.41 [4.96 to 5.86]	6.22 [5.75 to 6.69]	< 0.001	< 0.001	= 0.001
PSFS scores for the main categories						
Physical activity and exercise	2.83 [2.34 to 3.31]	4.99 [4.47 to 5.52]	6.13 [5.56 to 6.70]	< 0.001	< 0.001	< 0.001
Work and education	2.73 [2.11 to 3.36]	4.51 [3.83 to 5.19]	5.32 [4.60 to 6.03]	< 0.001	< 0.001	= 0.065
ADL activities	4.35 [3.71 to 4.99]	6.24 [5.54 to 6.94]	6.98 [6.24 to 7.73]	< 0.001	< 0.001	= 0.095
Social activities	3.47 [2.85 to 4.09]	5.46 [4.76 to 6.15]	6.12 [5.40 to 6.84]	< 0.001	< 0.001	= 0.135
Cognitive and energy functions	3.63 [2.98 to 4.28]	5.86 [5.14 to 6.58]	6.57 [5.83 to 7.32]	< 0.001	< 0.001	= 0.114
Activities in sensory demanding environments	3.15 [2.38 to 3.92]	5.73 [4.88 to 6.59]	5.86 [4.96 to 6.76]	< 0.001	< 0.001	= 0.827

Note: Linear mixed-effects model adjusted for age, sex, time since injury, and group, with a random intercept for participant. β = unstandardized regression coefficient; CI = confidence interval. PSFS total score model covariates: group (p = 0.727), age (p = 0.320), sex (p = 0.976), time since injury (p = 0.442). PSFS main category model: group (p = 0.621), age (p = 0.365), sex (p = 0.928), time since injury (p = 0.459).

Abbreviations: PSFS: Patient Specific Functional Scale, ADL: Activities of Daily Living.
